# Behavioral Attitudes and Preferences in Cooking Practices with Traditional Open-Fire Stoves in Peru, Nepal, and Kenya: Implications for Improved Cookstove Interventions

**DOI:** 10.3390/ijerph111010310

**Published:** 2014-10-03

**Authors:** Evelyn L. Rhodes, Robert Dreibelbis, Elizabeth Klasen, Neha Naithani, Joyce Baliddawa, Diana Menya, Subarna Khatry, Stephanie Levy, James M. Tielsch, J. Jaime Miranda, Caitlin Kennedy, William Checkley

**Affiliations:** 1 Division of Pulmonary and Critical Care, School of Medicine, Johns Hopkins University, Baltimore, MD 21205, USA; E-Mails: erhodes@jhsph.edu (E.L.R.); emklasen@gmail.com (E.K.); nvnaithani@gmail.com (N.N.); steph.a.levy@gmail.com (S.L.); 2 Department of Anthropology, University of Oklahoma, Norman, OK 73019, USA; E-Mail: rdreibe@ou.edu; 3 School of Medicine, College of Health Sciences, Moi University, Eldoret, Kenya; E-Mails: nabitajoy@yahoo.com (J.B.); dianamenya@gmail.com (D.M.); 4 Nepal Nutrition Intervention Project Sarlahi, Kathmandu, Nepal; E-Mail: skhatry@wlink.com.np; 5 Department of Global Health, School of Public Health and Health Services, George Washington University, Washington, DC 20052, USA; E-Mail: jtielsch@email.gwu.edu; 6 CRONICAS Center of Excellence in Chronic Diseases, Universidad Peruana Cayetano Heredia, Lima 31, Peru; E-Mail: jaime.miranda@upch.pe; 7 School of Medicine, Universidad Peruana Cayetano Heredia, Lima 31, Peru; 8 Program in Social and Behavioral Interventions, Department of International Health, Bloomberg School of Public Health, Johns Hopkins University, Baltimore, MD 21205, USA; E-Mail: caitlinkennedy@jhu.edu

**Keywords:** cookstove, household air pollution, resource-limited settings, behavior analysis, adoption, qualitative research, formative research, technology

## Abstract

Global efforts are underway to develop and promote improved cookstoves which may reduce the negative health and environmental effects of burning solid fuels on health and the environment. Behavioral studies have considered cookstove user practices, needs and preferences in the design and implementation of cookstove projects; however, these studies have not examined the implications of the traditional stove use and design across multiple resource-poor settings in the implementation and promotion of improved cookstove projects that utilize a single, standardized stove design. We conducted in-depth interviews and direct observations of meal preparation and traditional, open-fire stove use of 137 women aged 20–49 years in Kenya, Peru and Nepal prior in the four-month period preceding installation of an improved cookstove as part of a field intervention trial. Despite general similarities in cooking practices across sites, we identified locally distinct practices and norms regarding traditional stove use and desired stove improvements. Traditional stoves are designed to accommodate specific cooking styles, types of fuel, and available resources for maintenance and renovation. The tailored stoves allow users to cook and repair their stoves easily. Women in each setting expressed their desire for a new stove, but they articulated distinct specific alterations that would meet their needs and preferences. Improved cookstove designs need to consider the diversity of values and needs held by potential users, presenting a significant challenge in identifying a “one size fits all” improved cookstove design. Our data show that a single stove design for use with locally available biomass fuels will not meet the cooking demands and resources available across the three sites. Moreover, locally produced or adapted improved cookstoves may be needed to meet the cooking needs of diverse populations while addressing health and environmental concerns of traditional stoves.

## 1. Introduction

Globally, approximately 2.4 billion people rely on burning solid fuels (wood, dung, crop residue, garbage, or coal) for cooking, heating and lighting [1]. Combustion of these fuels releases smoke and toxins, such as carbon monoxide and particulate matter, that are associated with both acute and chronic negative health outcomes [2,3,4], including respiratory- and vision-related illnesses [1,5], heart rate variability [6], low birth weight and cancer [7]. Exposures to household air pollution are higher among women and children due to time spent cooking and tending indoor fires [3]. The World Health Organization has estimated that 1.5 million women and children living in the developing world die each year due to household air pollution [3]. Additionally, solid fuel burning stoves contribute to environmental degradation through deforestation and the production of greenhouse gasses [8].

Improved household cookstoves are increasingly positioned as an important strategy for reducing the negative effects of burning solid fuels on health and the environment [9,10,11,12,13,14]. Improved technologies are defined by decreasing household air pollution, improving fuel economy and user convenience [15]. Efforts by the World Health Organization have focused on making improved cookstoves widely available in low- and middle-income countries by 2015 [16]. Global partnerships, such as The Partnership for Clean Indoor Air and the Global Alliance for Clean Cookstoves, aim to reduce smoke exposure from cooking and heating practices [13]. The United Nations [14,17], the World Bank [18], governments in multiple regions of the world [19] and prominent public figures are promoting improved cookstoves and raising funds to implement new cooking technology [17]. These international efforts typically utilize standardized, pre-fabricated improved cookstoves that reduce emissions, increase efficiency of burning solid fuels and protect users from the negative health outcomes associated with household air pollution in resource-limited settings.

Existing research on improved cookstoves has generally focused on technology development or measuring the environmental and biomedical effects of burning solid fuels [4,20]. Adoption and utilization rates of improved stoves, even within highly controlled randomized trials, remain low [21]. Low durability of previous improved stove designs has resulted in stove abandonment in multiple settings [15]. Even with more robust modern designs, few studies have shown consistent adoption [15,22]. As a result, direct and indirect impacts of implementing improved cookstoves remain unknown [23,24]. A limited number of studies have specifically investigated factors affecting improved cookstove adoption and use in resource-limited settings [19,20,25,26]. Economic barriers to purchasing and maintaining non-traditional stoves have been shown to inhibit adoption [2,13,18,27,28,29,30,31]. However, even when financial burdens are lifted through subsidies or free distribution of stoves, potential users do not adopt or sustain exclusive use [21,30,32], suggesting that other, non-economic factors, such as user practices, needs, and preferences in relation to improved stove technologies [25,33] influence behaviors.

Researchers, product designers and implementing organizations have largely overlooked traditional cooking practices and traditional stove uses to understand the implication of these practices on potential improved stove adoption and utilization [25,34,35,36]. To increase adoption, improved cookstoves need to incorporate sociocultural acceptability, meet or surpass the user requirements of the traditional stove, and mitigate the environmental and biomedical impact of these traditional technologies.

Using multiple qualitative research methods embedded within a larger trial of improved cookstoves [37], we investigated how women in Peru, Nepal and Kenya use and view traditional, open-fire cookstoves in the pre-intervention period. We sought to characterize heterogeneity and identify commonalities in cooking practices and perceptions of traditional cookstove benefits and drawbacks among our three study populations. Moreover, observations of traditional stove use may suggest important areas for improved cookstove design and increased adoption. Utilizing theoretical models for the adoption of household environmental health technologies [38], we discuss the implications of our findings for the development of specific stove technologies, the implementation of improved cookstove programs and efforts to improve stove compliance.

Existing studies on cookstoves in each of our three countries have focused on economic barriers to adoption of improved technologies in Kenya and Nepal [2,31] and the health impacts of household air pollution in Peru [39,40,41]. Our research adds to the existing body of literature by describing traditional cooking practices and stove use in these three contexts and outlining the implications of these practices for improved cookstove adoption and stove design, both within the local context and other parts of the developing world [15].

## 2. Methods

### 2.1. Study Setting

The COCINAS trial took place in three rural, resource-limited settings in Kenya, Nepal and Peru [37]. The Uasin Gishu County in Western Kenya is located 2,200 m above sea level; wood and crop residues serve as the main sources of biomass fuel. The Sarlahi District in southern Nepal is 200 m above sea level, and >95% of the study population burns wood, dung and crop-waste for fuel. The Vinchos District in Ayacucho, Peru is 3500 m above sea level and residents burn wood or dung for cooking purposes. At all three sites, the majority of fuel burning takes place inside the home. This study was approved by the Institutional Review Boards of Universidad Peruana Cayetano Heredia (Lima, Peru), Johns Hopkins Bloomberg School of Public Health (Baltimore, MD, USA), Asociación Benéfica PRISMA (Lima, Peru), Institutional Research and Ethics Committee Moi University (Eldoret, Kenya) a branch of the National Council of Science and Technology (NCST), Institute of Medicine Tribhuvan University (Kathmandu, Nepal) and Lifespan/The Miriam Hospital (Providence, RI, USA).

### 2.2. Study Design and Data Collection

The COCINAS trial was a one-year study designed to examine environmental, biomedical and behavioral effects of traditional and improved cookstoves in rural, low-income settings. The study included one baseline and two consecutive intervention phases. Details on the larger study design are reported by Klasen *et al.* [37]. Qualitative data for this paper are taken only from the baseline four-month period of data collection assessing traditional, open-fire stove use to comprehensively understand existing behaviors and preferences prior to the intervention period. Following the observation and data collection periods reported in this paper, participants were randomized to receive one of two types of improved, ventilated cookstoves with a chimney: a commercially-constructed cookstove (Envirofit G3300/G3355; www.envirofit.org) and a locally-constructed cookstove. After four months of observation, participants will crossover and receive the other improved cookstove design for another four months. Following the initial four-month observation and data collection periods reported in this paper, participants were cycled through two types of improved cookstoves. Data from the post-intervention phase will be reported elsewhere.

Eligible participants were women between 20 and 49 years of age at the time of enrollment who: (1) used open-fire stoves; (2) were the primary cooks in their households, and (3) were willing to modify their homes to safely install two different improved cookstove designs over the course of the study.

#### 2.2.1. In-Depth Interviews

To gain an informed understanding of participant views on cooking with traditional stoves, we conducted in-depth interviews on two different occasions during the four-month period in Peru and Nepal, and once in Kenya. Trained data collectors interviewed participants in their local, native language. Interviews were recorded, transcribed, and translated into English. Interviews ranged from ten to thirty minutes in length, and covered topics such as:topics traditional stove use and maintenance, cooking practices, fuel collection and the impact of seasonal changes. We also asked the participants about their opinions of the traditional stoves, and perceptions of smoke and related illnesses.

#### 2.2.2. Direct Observations

In addition to the interviews, a series of direct observations of meal preparations were completed in each participant’s home. Trained field staff observed participants during morning and evening meal preparation, from the time of lighting the stove until the participant finished cooking, and recorded observations on the immediate cooking environment: lighting, ventilation, individuals present, fuel preparation and cooking practices. The data collectors observed all of the primary cook’s behaviors, including activities aside from meal preparation, and any apparent difficulties. The data collectors reported on smoke within the home and how the participants mitigated the immediate discomfort. Direct observations were completed twice during the four-month period in Peru and Nepal and once in Kenya and were conducted separately from the in-depth interviews.

#### 2.2.3. Data Analysis

We drew from grounded theory for our analyses, an approach to qualitative research that takes an iterative approach to data collection and analysis, and draws relevant themes from the data inductively rather than using existing theories to guide the research and analysis process [42,43]. For direct observation, emergent coding was used to identify key activities, events, and factors that shape cooking practices. These emergent, observation-based codes were then used to inform the organization and analysis of interview transcripts. Codes were further elaborated and developed to capture variability in practices and views of our study populations. This iterative review and analysis of the data was supplemented by structured sessions with in-country, local field staff who provided additional information on the socio-cultural contexts surrounding stove use and food preparation. Emergent thematic categories included: current cooking technologies, stove use patterns, individual perceptions of traditional stoves, and desired improvements.

In contextualizing our results, we utilized the IBM-WASH Framework [38], a composite framework based on multiple behavioral and ecological models intended to guide the design and evaluation of water and sanitation behavior change interventions that require some type of technological or hardware improvement. Similar to water and sanitation interventions, improved cookstoves are household-based technological interventions that attempt to modify and/or replace existing behaviors and practices.

## 3. Results

### 3.1. Participant Characteristics

Each of the three sites aimed to enroll up to 46 women at baseline to achieve an expected 40 participants per site followed throughout the trial. A total of 137 participants were enrolled at baseline for participation across all sites. Average age for the participants in Kenya, Peru and Nepal were 35.8 (SD = 6.6), 36.4 (SD = 7.8) and 37.3 (SD = 7.9) years of age, respectively (ANOVA test for differences between sites *p* = 0.62). The average household size was 5.4 people across the entire sample. Participants had an average of 3.7 children. Households were larger in Kenya (average 4.6 ± 2.2 children) than Peru (average 3.5 ± 1.6 children) or Nepal (average 3.1 ± 1.6 children; ANOVA test for differences between sites *p* < 0.001). Participants in Kenya had completed an average of 9.6 years of schooling (SD = 2.5) compared to 2.9 years in Peru (SD = 2.9) and 1.3 years in Nepal (SD = 3.4; Kruskal-Wallis test for differences between sites *p* < 0.001). All communities had agriculture-based economies, and participants at all sites maintained their livelihood by selling livestock, crops or dairy products.

### 3.2. Existing Technology

**Figure 1:**
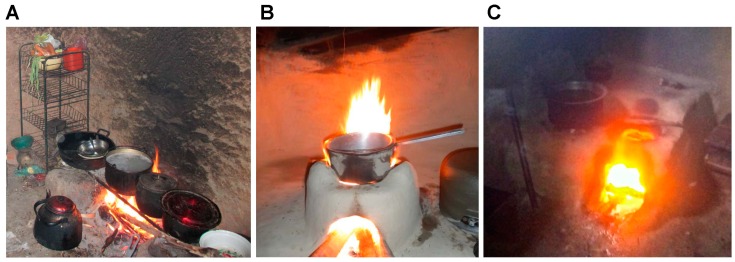
Traditional, open-fire, biomass burning cookstoves in Peru (**A**); Nepal (**B**) and Kenya (**C**).

Traditional stoves used by study participants were homemade, built on the ground and used locally available materials only. Aside from that, however, traditional stove designs varied greatly. In Peru, the *tullpa* typically consisted of two to three mud and clay based walls surrounding a fire pit with metal bars across the top (Figure 1A). Women often had one stove inside the kitchen and another outside. The Nepalese *maato chulho* was built from rice husks and clay, and varied in design depending on family size (Figure 1B). As in Peru, households typically had multiple stoves, both inside and outside the home. Kenyan households typically had only one stove for cooking, the *chepkube*, made from mud and clay (Figure 1C). They also used a small, portable coal-burning stove, known as the *jiko*, solely for warming purposes. The defining characteristic of the *chepkube* was a small chamber adjacent to the fire pit for warming food.

Participants in all three countries frequently explained that the traditional stoves represent fundamental part of their customs and ancestral heritage. One Peruvian woman stated, “Our grandparents cooked with this *tullpa*. That’s why we also continue to [use it]; because there is no other way”. The warming chambers observed on the *chepkube* served both a practical and symbolic function to Kenyan participants, all of whom were members of the majority Kalenjin tribe. As one participant stated, “Almost the entire area uses this type of fireplace. It is something that we were born while it was in use… and it is still in use”. Traditional stoves were thought to prepare the food properly, and yield the right flavors according to their custom. Participants who had experience using gas stoves in Peru complained that gas-prepared food lacked taste. In describing the use of her current stove, one Peruvian participant said “(open-fire stoves) cook quickly; it (food) tastes better than (with) gas”. The food, including the stove used to prepare it, was more desirable when associated with the traditional cooking and preparation practices.

### 3.3. Stove Use Patterns

The most frequently prepared meals in Peru, Nepal, and Kenya were carbohydrates: potatoes, rice and *ugali* (maize meal), respectively. Different requirements for heat distribution and stove stability in the preparation of each of these staple foods were reflected in the local open-fire stoves used at each site. Women in Nepal boiled rice in large metal pots without stirring; they required an even heat distribution to avoid burning the rice, but did not need a firmly grounded stove to withstand motion. As such, flames from the *maato chulho* were distributed over a wide area through a large burner opening for cooking. In Kenya, on the other hand, the *ugali* was cooked with rigorous, aggressive stirring; an even heat distribution was not necessary, but ability to sustain forceful movement was crucial. The *chepkube*, as such, was built into the ground, with low walls and a wide base. In Peru, women boiled potatoes without agitating the water. Therefore, neither heat distribution nor stove stability were major concerns for the staple dish.

The primary cook was consistently responsible for gathering and preparing fuel in all three countries. The type of fuel, however, varied between and within sites, and even within households depending on availability. In Peru, women preferred to burn wood, but most could not afford to purchase or collect enough firewood to last all year. Additionally, due to high altitudes, wood was a scarce commodity. Instead, they burned cow, sheep, and alpaca dung in the dry season, when it was easily harvested, and firewood during the rainy season. In Kenya, women only used firewood and crop residue for fuel; burning dung was considered offensive to the cattle and Kenyan participants thought it could reduce milk production. In Nepal, women burned firewood, dung, crop residue, and coal depending on what was available seasonally. Across sites, women appreciated the ability to leave large logs on the fire and keep the embers warm all day.

In all sites, stoves varied from house to house within each community. The most notable indicator for type of traditional stove type was socioeconomic status. Not surprisingly, women with more wealth and higher status had stoves with stronger walls, and even adornments, making them more durable and desirable because of the infrequent maintenance required. Women with fewer resources often cooked on completely open fires, or stoves with crumbling walls.

In Peru and Nepal, where women cooked on multiple stoves, various factors determined which stove was used at any given time. In Peru, weather conditions, meal type, fuel type and quantity of food being prepared determined which of the household’s stoves were in use. For example, toasted grains, fodder, and holiday dishes were usually prepared outside because they require more space. Most other meals, however, were prepared inside, protected from the wind, rain, and cold. Weather was a major factor in determining stove use in Nepal. In monsoons and during stormy or windy weather, most participants reported using indoor stoves. In peak summers, the outdoor stove was preferred to cooking over the uncomfortably warm indoor kitchen. Using crop residue as fuel also necessitated cooking outdoors for many participants because of the large amounts of sparks and ash generated. The non-vegetarian content also dictated where participants cooked, as meats are considered “impure” and have to be handled separately. This was especially relevant during festivals and religious ceremonies, where families built a temporary stove as a general cleansing and purification process and for cooking special, pure vegetarian foods.

Cleaning and maintenance of the stoves varied greatly between sites and was largely dependent on the type of fuel used and the amount of ash produced. With animal dung and garbage ash, participants reported cleaning their stoves daily, sometimes even after each use. With firewood, women reported cleaning their stoves weekly. Nepalese women also rebuilt and maintained their stoves for religious reasons; they aimed to appease and celebrate the goddess of prosperity and wealth Lakshmi for whom the stove was a temple. “If I do not clean and plaster it (the stove), (my) hands and legs might get burnt”, reported one Nepalese participant.

In all three sites, women reported utilized ash for a variety of practical and symbolic functions: Nepalese participants used it for cleaning utensils, Peruvian participants used it for fertilizer and for preparation of some traditional meals, and Kenyan participants used it to cover wounds on animals and treat respiratory illnesses by mixing it with water and drinking it. Depending on the fuel burned, ash produced had different value. Ash from dung was typically disposed of or used for fertilizer, while ash from firewood was used for cooking and cleaning. Ash accumulation on the ceiling from the smoke was used for medicinal purposes.

Stove use was not restricted to cooking food, although this practice also varied across sites. In Kenya and Peru, women heated their homes and warmed water for cleaning and bathing with their stoves. Fires were left unattended for long periods of time after the meal was completed in order to warm the home or keep food warm for absent family members. Women enjoyed the benefit of being able to leave their stoves unattended, but still keep their kitchens warm. Women in Nepal, however, did not report using their stoves for anything other than cooking.

Stove use and cooking was done within the larger context of domestic responsibilities and activities that women were responsible for accomplishing. Women in Peru, Nepal, and Kenya had an immense amount work do to throughout the day. In all three countries, women gathered fuel and prepared food while also tending to animals and crops, caring for children, and cleaned their homes and clothing. In Peru, the women also often prepared cheese or crocheted belts to sell in the markets. As a result, participants were constantly busy, with little room for leisure or additional responsibilities.

### 3.4. Perceptions of Stoves

Across sites, participants reported that they were pleased with the existing stoves’ suitability for burning local fuel, cooking for large families, and properly preparing traditional meals. Additionally, participants at all three sites appreciated the ability to leave their stoves and accomplish other tasks while cooking. One Kenyan participant explained, “many people in this area have (the traditional stove) and they like it because of the *chepkube* which enables you to cook and leave”.

Women also expressed frustrations with the traditional stove design. The amount of smoke emitted by the traditional stove was a main complaint among Kenyan and Peruvian participants, but most Nepalese did not refer to the smoke as a disadvantage. One participant asked, “How can the smoke coming out of the stove harm you”? Across all study participants, women had differing perceptions of the risk of household air pollution produced by their traditional stoves. In Peru and Nepal, most perceived the smoke to only cause immediate discomfort. In Kenya, however, participants expressed concern for long-term health effects. Participants in Kenya and Peru referred to smoke from their traditional stoves as something to which one grows accustomed, since exposure is inevitable. This attitude was expressed about many inconveniences faced by the communities in all three sites, such as cold or rainy weather.

Routine renovations and frequent reinstallations, tasks expected to be completed by men in some settings and women in others, were the most common areas of dissatisfaction participants articulated with regard their traditional stove. In all sites, women both were observed and reported smearing and patching cracks in the mud frequently, and they often renovated or reinstalled the stove entirely. Women in Peru complained of the heat burning through the stove walls and bending the metal bars, forcing them to replace materials. Rebuilding the stove took time and resources, and when participants neglected to maintain their stoves, they could become dangerous and inefficient. Most women found the constant need for maintenance to be a major inconvenience. Many participants in Peru left their stoves in disrepair for extended periods of time. Across sites, the ability to reconstruct stoves using local materials meant that for minor breaks, women could easily and quickly repair their own stoves. One woman in Nepal stated, “(I) replace the mud of the stove when I feel like it, and the stove becomes (like) new. It is my own wish whether I want to change my stove in two or three months or keep it for one whole year by changing the clay”. Having the freedom to easily fix or modify the stove was a valuable quality.

### 3.5. Desired Improvements

Participants unanimously expressed hopes for new a stove design. The priorities of what to improve, however, varied for each country. In Nepal, participants expressed that their husbands’ satisfaction the meal preparation time was an important factor influencing stove use. If the cooking time took too long, some women reported that they would experience verbal or physical abuse. They expressed that “whoever is cooking *roti* needs to be scared” for fear of the consequences of a delayed meal. Therefore, they wanted a stove with the capacity to cook for many people, quickly.

In Kenya, participants articulated a strong dislike for mud-based cookstoves. One participant explained how inconvenient it could be: “the mud, you go for it from the far distance… sometimes (you) have a lot of work”. They said that mud was difficult to gather in dry seasons and that mud-based stoves are in constant need of repair. Women even threatened to leave the trial if they received a mud-based improved cookstove.

In Peru, participants generally desired more durable stoves with chimneys to reduce smoke exposure. One woman stated, “I would want a pretty *tullpa*, straight and tall. With a chimney so that the smoke goes out through it”. In other countries, however, few participants identified smoke or reductions in smoke as something they wanted to change about their current stoves.

There were several existing barriers to individuals improving their existing stoves. Many participants said they used their traditional stoves out of necessity and lack of other options. One participant articulated this lack of alternatives: “how are we going to make our food if not in (that) stove”? Women also referred to their lack of knowledge and means of accessing other methods as reasons to use the traditional stove.

## 4. Discussion

Our initial four-month observation period investigated cooking practices and the use of traditional open-fire stoves in Peru, Nepal and Kenya to characterize both differences and similarities in practice. We used methodological triangulation to combine insights from both direct observations and multiple interviews to develop a better understanding of how women use and perceive their stoves. We used both direct observations and interviews at different times to better understand participant behavior. This approach helped us characterize local opinions, potential reactions to change, and the type of improvements based on participants’ desires. Findings from this study demonstrate the effect of contextual factors, background characteristics that cannot be changed by an intervention, on cooking behaviors as has been reported in other studies [22,23]. Wide variability between sites in regard to habits and attitudes towards traditional stove use suggests that a single improved stove design using locally-available biomass fuels may not meet the diverse needs in resource-limited settings [26]. We discuss these differences and their implications for improved cookstove design and promotion according to the three dimensions of the IBM-WASH Framework: technological factors, psychosocial factors, and contextual factors. Based on the specifications in regard to stove use and resources available, improved technologies are most likely to be adopted and used if they are modified to be more efficient and safe while maintaining positive characteristics of traditional designs.

### 4.1. Technological Factors

Studies have shown that inappropriate improved cookstove designs, such as inconvenient stove size and instability, prevent women from adopting new stoves [44,45,46]. In this study, data confirmed that convenient technological design and logistical ease of use are important factors in current stove use. We noted high variability in traditional stove design both between and within each site, and often within the same household as has been found in previous studies [25]. Since the stoves were built entirely from local, easily accessed materials, women could easily alter their stoves. Although repairs were frequent, the method of repairing the traditional stove was usually quick and easy. The practicality of conveniently produced or manufactured materials presents a significant benefit to the traditional stove, and potential barrier to any improved cookstove that relies on a complex design or imported materials. There is conflicting literature on the role of complexity and designs and its relation to adoption and use. In Nepal, Pandey and Yadama [47] found that complex improved stoves did not deter adoption; while participants in a study in Mexico complained about the difficulties in growing accustomed to new, complicated technology [30].

The incorporation of new technologies into the domestic sphere is often associated with marked in increases in time and effort needed to maintain and operate newly introduced technologies [48]. Reduced maintenance for any stove was highly desired among participants, and improved stove design should be easily renovated in order to foster adoption. Cookstove trials demonstrate that designs requiring scheduled cleaning of the flue and combustion chamber were often inadequately maintained [30].

A single traditional stove, built according to local customs and traditions, was often insufficient to meet a household’s cooking needs—either practically or due to cultural restrictions on food preparation. Designing new technology to replacing all user needs with one, single stove would be beyond the potential of even traditional designs, tailored specifically to their practices and traditions. Studies have noted a diversity of cooking sources within the household even during controlled trials in which new, improved stoves were provided [21,25,45]. Continued use of traditional stoves during improved stove evaluation impacts the findings, and ultimately reduces any benefit from using the new stove. Based on previous studies and the formative data collected in this trial, designing a single stove to meet the needs of all users risks obligating users to continue using traditional open fires and continued exposure to household air pollution.

The practicality of ash produced with traditional stove users presents a dilemma for improved cookstove design. Ash and combustion by-products were valuable commodities for many of our participants. Improved cookstove designs that promote more efficient fuel burning will, by default, limit availability of these resources. Additionally, using a chimney vented to the exterior interferes with the production of ash stalactites, a substance used for traditional remedies for respiratory illness in Kenya. The loss of this local approach to illness management should be considered in cookstove promotion programs. The novel application and economic and symbolic role that ash and ash production carries in cooking has overlooked in existing research on cookstoves and could be a potential deterrent to adoption in more traditional settings.

With traditional cookstoves, constructed to fit cooking utensils and pots, and to prepare the customary dishes, women can easily, effectively, and quickly prepare their food. Variation in stove design, described above, reflects the unique cooking needs of each population. Increased cooking time with improved cookstoves has been found to be a deterrent to adoption [45,49]. Further, increased cooking time may have unintended consequences in the domestic space. Women in Nepal reported incidence of domestic violence against women who are unable to prepare meals in a timely manner. Flexible stove designs that respond to local cooking practices are needed to ensure widespread adoption and use of improved cookstove technologies.

### 4.2. Psychosocial Factors

Women in all sites agreed that maintaining tradition was a relevant benefit of using their traditional stoves and that cooking and specific food preparation practices were a component of their cultural identity. A single, universal stove design may not meet the cooking demands and local practices across different settings. Aspirations for a new stove varied between sites, depending on social norms and contextual needs (such as available resources) for each site.

Low perceived risk of smoke and smoke exposures has been identified in a limited number of studies [18,28,50]. In reference to household air pollution from traditional stoves, our participants generally had little knowledge of the health risk associated with exposure to smoke and perceived the risk of these threats as very low. Observation data show respondents reacting to the immediate consequences of smoke: coughing, stinging of the eyes, and choking; but none of our respondents linked these exposures to long-term negative health impacts. Particularly in Peru and Kenya, smoke exposure was viewed as an inevitability that one grows accustomed to rather than an irritant that should be mitigated.

Previous studies have demonstrated that education about the detrimental impacts of household air pollution, combined with marketing and availability foster behavior change [18,28,29,47,51]. Further research is necessary to understand the known and perceived risk of household air pollution among traditional stove users. That data will improve efforts to market new technologies and educate women about the benefits of improved cookstoves.

### 4.3. Contextual Factors

The contextual factors are those related to the larger social and physical environment in which behaviors are practiced. We noted large fuel variability within individual participant homes during the observation period. Rather than a reflection of personal choice, fuel use was typically driven by available resources. The variability places an advantage on those stoves that accommodate multiple fuel types rather than improved stove designs that require specific combustion products. Many improved designs are meant to burn any fuel, from garbage and crop residue to firewood and coal. However, although the design allows for flexibility in fuel type, method of fuel preparation and fire maintenance are limited due to other design factors. The openings of improved stove combustion chambers are too small to add a large log to burn all day, as most women prefer. Additionally, most improved stoves require adding fuel from the side of the combustion chamber, instead of from the top, making it difficult to add small pieces of fuel (dung or garbage) without extending the user’s hand into the fire.

Women have a number of responsibilities within the domestic environment, and they have little room for adjustment within their routine. Adding to their workload, whether in energy spent preparing fuel or time dedicated to cooking, would be a burden to their already busy lives. A change in technology requiring new chores, such as longer fuel preparation, or more intensive fire maintenance, would prevent women from accomplishing their other responsibilities.

Although cost was not a relevant part of this study, data show that wealthier participants with comfortable homes, employed family members, and sufficient resources consistently had higher quality stoves. In Mexico, one study found that socioeconomic level was positively correlated with improved cookstove adoption [30]. A study in Tanzania found that cost was a barrier in the adoption of improved stove technologies [29]. Therefore, although cost was not a factor of this study, data are consistent with previous research implying that wealth impacts the quality of stoves that households utilize.

### 4.4. Potential Limitations

Since participation in the larger trial was voluntary, our findings may represent the perspectives of women who already desired new stoves. Participants also knew that data collectors interviewing them would provide new, locally and internationally improved stoves at a later phase in the study. Therefore, courtesy bias, where responses are tailored to please the data collectors and the larger objectives of the research rather than reveal the participants’ true opinions, might impact data accuracy. Although participants were reassured that they would receive new stoves regardless of their responses, data collectors suspected that participants reported dislike toward their traditional stove to ensure they would receive a new one.

Although all data collectors were bilingual, nuances in language could have been lost during the process of translation from the native language to the national language to English. Additionally, due to logistical complications, such as road condition and weather, we were unable to conduct two interviews and observations in Kenya within the four-month period.

## 5. Conclusions

A number of technological, psychosocial and contextual factors influenced the perceptions of traditional cookstoves. Understanding attitudes and practices toward traditional stoves can impact stove design and implementation and ultimately increase adoption [19,22,26,36]. The findings from this study reveal nuances of traditional stove use in three diverse settings in Peru, Nepal and Kenya. We found that although there were many similarities across the three sites, women from each country employed vastly different cooking methods and in response to variations in fuel availability, cooking requirements, and existing local practices. Women used multiple traditional stove constructions and settings to meet their cooking needs, even within the same kitchen.

The behaviors and preferences associated with traditional cookstove use undoubtedly play an important role in determining the adoption rates of improved cookstove interventions [22,36]. Therefore, future improved cookstove designs should consider the diversity of these issues, and heterogeneity in local cooking practices presents a significant challenge to designing a single stove that meets the needs of users across resource limited settings. Therefore, an improved cookstove design and implementation based on intended users’ needs would have to be setting specific. Understanding local cooking needs, practices, and traditions may inform the design and development of new stoves that meet the needs and behaviors of target populations. In Peru, Nepal and Kenya, the characteristics that determine logistical convenience varied, but most women appreciate large, stable stoves that are easily maintained. Women from each site prefer stoves that can cook their traditional foods well in a traditional manner. Women from different setting require different materials and fuels for the stoves, different methods of ash removal. Women perceive the risk of smoke inhalation differently, and would therefore require different educational and promotional materials in dissemination of improved technologies. Although the findings imply that one, universal stove cannot meet the needs across settings; they reveal important issues that need to be taken into consideration.

Accommodating the specific fuel type, style of food preparation, and user needs and preferences within the context of each setting would facilitate adoption and therefore effectively reduce negative health outcomes associated with exposure to household air pollution. In conclusion, to increase adoption rates, improved cooking technologies should be designed based on traditional stoves with modifications to reduce biomedical and environmental harm.
